# Incidence of Visually Impairing Cataracts Among Older Adults in Kenya

**DOI:** 10.1001/jamanetworkopen.2019.6354

**Published:** 2019-06-28

**Authors:** Andrew Bastawrous, Wanjiku Mathenge, John Nkurikiye, Kevin Wing, Hillary Rono, Michael Gichangi, Helen A. Weiss, David Macleod, Allen Foster, Matthew Burton, Hannah Kuper

**Affiliations:** 1 International Centre for Eye Health, Clinical Research Department, London School of Hygiene & Tropical Medicine, London, United Kingdom; 2 Rwanda International Institute of Ophthalmology and Dr Agarwal’s Eye Hospital, Kigali, Rwanda; 3 Department of Non-Communicable Disease Epidemiology, London School of Hygiene & Tropical Medicine, London, United Kingdom; 4 Kitale Eye Unit and Trans Nzoia County, Kitale, Kenya; 5 Ministry of Health, Nairobi, Kenya; 6 MRC Tropical Epidemiology Group, Department of Infectious Disease Epidemiology, London School of Hygiene & Tropical Medicine, London, United Kingdom; 7 Moorfields Eye Hospital, London, United Kingdom

## Abstract

**Importance:**

Half of all the cases of blindness worldwide are associated with cataract. Cataract disproportionately affects people living in low- and middle-income countries and persons of African descent.

**Objective:**

To estimate the 6-year cumulative incidence of visually impairing cataract in adult participants in the Nakuru Eye Disease Cohort Study in Kenya.

**Design, Setting, and Participants:**

This secondary analysis of the Nakuru Eye Disease Cohort Study was conducted from February 2016 to April 2016. This cohort comprised citizens of Nakuru, Kenya, aged 50 years or older who consented to participate in the initial or baseline survey from January 2007 to November 2008, as well as the follow-up conducted from January 2013 to March 2014. All participants at baseline (n = 4364) and follow-up (n = 2159) underwent ophthalmic examination.

**Main Outcomes and Measures:**

Six-year cumulative incidence of visually impairing cataract, risk factors of incidence, population estimates, and required cataract surgical rates to manage incident visually impairing cataract.

**Results:**

In total, 4364 individuals (with a mean [SD] age of 63.4 [10.5] years and with 2275 women [52.1%]) had complete eye examinations at baseline, and 2159 participants (with a mean [SD] age of 62.5 [9.3] years and with 1140 men [52.8%]) were followed up 6 years later. The 6-year cumulative incidence of visually significant cataract in either eye was 251.9 per 1000 (95% CI, 228.5-276.8), with an increase with age from 128.9 (95% CI, 107.9-153.2) per 1000 for the group aged 50 to 59 years to 624.5 (95% CI, 493.1-739.9) per 1000 for the group aged 80 years or older. This equated to an annual incidence of visually significant cataract of 45.0 per 1000 people aged 50 years or older. Multivariable analysis showed alcohol consumption (risk ratio [RR], 1.4; 95% CI, 1.1-1.8), diabetes (RR, 1.7; 95% CI, 1.3-2.3), educational level, and increasing age (RR, 3.8; 95% CI, 2.6-5.5 for those aged ≥80 years) were associated with incident visually impairing cataract. Extrapolations to all people aged 50 years or older in Kenya indicated that 148 280 (95% CI, 134 510-162 950) individuals might develop new visually impairing cataract in either eye (visual acuity <6/18 in the worse-seeing eye) and that 9540 (95% CI, 6610-13 750) might become cataract blind in both eyes (visual acuity <3/60 in better-seeing eye).

**Conclusions and Relevance:**

Adults in Kenya appeared to have a high incidence of visually impairing cataract, making cataract a priority for blindness prevention programs in the region; surgical interventions and awareness of these services are also required.

## Key Points

**Question:**

How many new people per year become visually impaired from cataract in Kenya?

**Findings:**

In this secondary analysis of the Nakuru Eye Disease Cohort Study of 4364 participants at baseline and 2159 participants at follow-up, the 6-year cumulative incidence of visually significant cataract in either eye was 251.9 per 1000, with the incidence increasing with age among those aged 50 to 59 years and those 80 years or older.

**Meaning:**

In Kenya, reducing the burden of sight loss from cataract is a national priority, given its high incidence among older adults; the cataract surgical rate needs to be at the level of the incident rate to prevent the prevalence of blindness and visual impairment from increasing.

## Teaser

This secondary analysis of the Nakura Eye Disease Cohort Study estimates the 6-year cumulative incidence of visually impairing cataracts among older adults in Kenya.

## Introduction

The prevalence and incidence of cataract are known to increase with advancing age, and the magnitude of visually impairing cataract is expected to continue to grow with the aging populations and longer life expectancies worldwide.^1^ Half of all cases of blindness worldwide are associated with cataract.^2^ Cataract disproportionately affects people living in low- and middle-income countries and persons of African descent.^2,3^ Multiple population-based studies have been conducted of the prevalence of cataract in sub-Saharan Africa,^4^ and they have found a considerable variation in prevalence across the continent. However, surveys have routinely shown that cataract is the condition most associated with blindness or visual impairment in sub-Saharan Africa.^4^

Previous studies of the overall incidence of blindness and visual impairment,^5^ macular degeneration,^6^ diabetes and diabetic retinopathy,^7^ and glaucoma^8^ used data from the Nakuru Eye Disease Cohort Study. This cohort study of adults aged 50 years or older living in Nakuru, a city in the Rift Valley region in Kenya, was deemed to be a regionally and nationally representative sample that could inform the eye care needs and priorities of the entire country. The same cohort study serves as the source for this current analysis, which characterizes the incidence of visual impairment associated with cataract.

Management of cataract involves the surgical removal of the lens and insertion of an intraocular lens and is considered one of the most cost-effective health interventions worldwide.^9^ Identifying the cataract surgical rate needed to control the cataract blindness rate depends on estimating the incidence of cataract. However, the only incidence data on cataract from populations of African descent come from outside the African continent. The best estimates come from the Barbados Eye Studies,^10,11,12,13^ a 9-year follow-up of adults of African descent aged 40 years or older, showing incidence rates of 33.8% for any cortical opacities and 42.0% for any nuclear opacities and indicating these rates were higher in participants of African descent than those of white or Caucasian race/ethnicity (risk ratio [RR], 1.8; 95% CI,1.2-2.8).^13^ Incidence data are urgently needed for Africa to ensure appropriate planning and allocation of scarce human resources and equipment.

In this present secondary analysis, we aimed to estimate the 6-year cumulative incidence of visually impairing and blinding cataract among participants in the Nakuru Eye Disease Cohort Study. This cohort comprised people of East African ethnicity aged 50 years or older who lived in Nakuru, Kenya.

## Methods

The methods of the Nakuru Eye Disease Cohort Study have been reported in detail previously,^14^ are summarized here, and appear in the eMethods of the Supplement. The present study, conducted from February 2016 to April 2016, followed the Strengthening the Reporting of Observational Studies in Epidemiology (STROBE) reporting guideline. It adhered to the tenets of the Declaration of Helsinki^15^ and was approved by the Ethics Committee of London School of Hygiene & Tropical Medicine at both baseline and follow-up surveys.

The objectives of the Nakuru Eye Disease Cohort Study survey and the examination process were explained in the local dialect to eligible participants in the presence of a witness. A participant underwent examination only after written (or thumbprint) informed consent was obtained. Participants identified with eye or other medical conditions were referred to local health care services.

The initial or baseline population-based survey was conducted from January 2007 to November 2008. The sample size of 5000 participants aged 50 years or older was calculated according to an expected prevalence of visual acuity (VA) less than 6/12 (Snellen equivalent) in the better eye owing to posterior segment eye diseases (the primary outcome for the baseline survey) of 3.0% in this age group, precision of 0.5%, design effect of 1.5%, and a response rate of 90%.

One hundred clusters of 50 participants each were selected, with a probability proportional to the size of the population across Nakuru district. Households were selected within clusters, using a modified compact segment sampling method.^16^ An eligible individual was someone aged 50 years or older living in the household for at least 3 months in the previous year. All participants were invited to undergo a comprehensive ophthalmic examination at a screening clinic. The follow-up survey of the cohort was conducted from January 2013 to March 2014.

### Baseline and Follow-up Examination Clinics

The following procedures were undertaken for all participants who attended the examination clinic at baseline and follow-up surveys, and further details are available elsewhere.^14^ Additional procedures were undertaken that are not included here because they are not relevant to the outcomes (eg, visual field assessment) being reported.

On examination day, the advance team confirmed the identity of participants against baseline data (ie, age, date of birth, name, and identity cards). In cases of uncertain identity, confirmation was made by retinal examination verified by comparison with the baseline photo.

A clinical officer assessed whether study participants wore distance correction glasses, owned distance correction glasses but failed to bring them, did not own any distance correction glasses, routinely used reading glasses, or wore aphakic glasses. Visual acuity was measured using a back-illuminated modified logMAR reduced tumbling E chart (Sussex Vision Inc),^17,18^ which has been used in previous population-based studies.^19,20^

The following vision categories were used to define eye-level and person-level (based on the better-seeing eye) VA: normal (≤6/12 Snellen; logMAR ≤0.3), mild visual impairment (VI; <6/12 to 6/18 Snellen; <0.3 to 0.48 logMAR), moderate VI (<6/18 to 6/60 Snellen; <0.48 to 1.0 logMAR), severe VI (<6/60 to 3/60 Snellen; <1.0 to 1.3 logMAR), or blind (<3/60 Snellen; <1.3 logMAR). The term *visually impaired* was used to describe participants with a VA less than 6/18 to no perception of light and therefore included moderate VI, severe VI, and blind.

Pharmacologic dilation of the participant pupils was achieved by using tropicamide, 1% (Mydriacyl; Alcon Laboratories Inc), with phenylephrine hydrochloride, 2.5%, if needed. The anterior segment was examined by the study ophthalmologist (W.M. at baseline; A.B. at follow-up) using slitlamp biomicroscopy. The World Health Organization Simplified Cataract Grading System was used^21^ following standard protocols. The lens was also examined for position, the presence of hypermature (morgagnian) cataract, and previous lens (aphakic or pseudophakic) surgical procedure. A red reflex lens image was taken when each participant took their retinal photographs. Participants who were pseudophakic were assessed for the presence or absence of posterior capsular opacification and, if present, whether it entered the visual axis.

Visually impairing cataract was defined as VA in the better-seeing eye of less than 6/18 and the presence of a gradable cataract (nuclear, cortical, posterior capsular, or mixed, according to the Simplified Cataract Grading System^21^), mature cataract, or hypermature cataract. Definitions of incidence are found in eTable 1 in the Supplement.

All participants who had complete examinations at baseline and were not classified as having a visually impairing cataract were considered to be at risk for incident visually impairing cataract. Follow-up status at 6 years was categorized as (1) found and examined, (2) found and not examined, (3) deceased, (4) moved away, or (5) unknown.

### Statistical Analysis

Statistical analysis was performed with Stata, version 13 (StataCorp LLC), from January 2015 through July 2015. All analyses accounted for the cluster survey design using Taylor linearized variance estimation to calculate SEs. Pearson χ^2^ tests corrected for the survey design were used to calculate 2-sided *P* values to assess differences between participants seen and participants lost to follow-up as well as between those known to have died and those with unknown outcome status.

Participants who died and therefore did not have outcome data were excluded, as they were not eligible for follow-up. Participants who were followed up but had no complete records for all covariates at baseline were also removed from the cohort at this stage. An inverse probability weighting model^22^ was developed to allow estimation of cumulative incidence while accounting for participants lost to follow-up. Multivariable logistic regression was used to identify independent baseline covariates associated with lost to follow-up. Covariates with evidence of univariable association with the outcome (*P* < .10 across all categories of the variable) were kept in a multivariable model, whereas those with *P* > .10 were excluded from the model. From this final model, the probability of being followed up was estimated on the basis of the presence or absence of each of these baseline covariates. The inverse of this probability formed the weighting to be applied to account for those lost to follow-up.

The final step was to remove those individuals lost to follow-up from the cohort so that all subsequent analysis would be performed on only those with complete outcome records, with inverse probability weighting applied to account for those lost to follow-up. A sensitivity analysis for this approach involved a complete records analysis (ie, only including people who had complete records for outcome and all variables).

The 6-year cumulative incidence of each outcome was calculated by dividing the number of events identified at the 6-year follow-up by the number of people at risk at the beginning of follow-up. We estimated 95% CIs, assuming a Poisson distribution of events. This step was done for the population overall, which was stratified by each covariate.

To estimate age-adjusted associations between each outcome (VI and blindness), with baseline covariates, we calculated age-adjusted RRs for each covariate using a Poisson regression model with robust error variance to allow for the clustered design and including inverse probability weighting. For multivariable analysis, an initial model was fitted that included those variables shown to be associated with outcome in age-adjusted analysis (using a Wald test threshold *P* < .05 to indicate association). A backward approach was then applied to obtain a final multivariable model, removing one by one the variables with *P* > .05.

World Health Organization definitions of VI and blindness were used throughout^23^: monocular VI was VA less than 6/18 (20/60) in either eye, VI was VA less than 6/18 in the better-seeing eye, and monocular blindness was VA less than 3/60 (20/400) in either eye. A person was considered blind if the VA in the better-seeing eye was less than 3/60. The definition of VI also included those who were blind.

Diabetes was defined as (1) self-reported in the history, (2) random glucose level of 198.2 mg/dL or higher (to convert to millimoles per liter, multiply by 0.0555), or (3) HbA_1c_ percentage of total hemoglobin level of 7.0 or higher (to convert to proportion of total hemoglobin, multiply by 0.01).

Estimates of cumulative incidence were extrapolated to estimate the number of adults older than 50 years with incident VI or blindness in Kenya. The 2015 Census Bureau of Kenya population estimates were identified by age category and sex and then multiplied by the age- and sex-specific estimates of annual cumulative incidence.

The number of cataract surgical rate (CSR) per million of population (all ages) was estimated at different surgical thresholds on the basis of 3 levels of VA (blind, severe VI, or moderate VI) and whether for person or for individual eye. The estimated annual CSR per million of population was calculated by multiplying the annual incidence rate for all aged 50 years or older by 1000 and by the proportion of the population aged 50 years or older in Kenya (4.3 million of 45 million in 2015). The CSR calculation assumed no cases of blinding or visually impairing cataract existed among people younger than 50 years and was therefore likely to underestimate the true incidence by a small amount.

## Results

### Estimates of Prevalence

In total, 4414 participants were recruited at the baseline survey in 2007 to 2008. Of these participants, 4364 (98.9%; with a mean [SD] age of 63.4 [10.5] years and with 2275 women [52.1%]) had an examination of the lens and were given a lens status. Among the 4364 individuals who had complete eye examinations, 669 (15.3%) had VA less than 6/12 in the better-seeing eye. Of these 669 participants, 180 (26.9%) were visually impaired (VA <6/18) from cataract, with 32 of them blind, 11 with severe VI, and 137 with moderate VI.

Cataract was the most commonly associated with blindness, affecting 1968 participants (45.1%), and severe VI, affecting 2666 participants (61.1%). Overall, 3591 participants (82.3%) did not have VI or visually significant cataract; that is, they had no cataract and VA of 6/18 or better, had the presence of cataract but VA of 6/18 or better, or had VA of worse than 6/18 but no evidence of cataract (Table 1).

**Table 1.** Cumulative Incidence of Visually Significant Cataract Among Study Participants

Age Group, y	Male		Female		Overall	
	Cases/at Risk, No.	Risk per 1000/6 y (95% CI)	Cases/at Risk, No	Risk per 1000/6 y (95% CI)	Cases/at Risk, No.	Risk per 1000/6 y (95% CI)
**Either-Eye Cataract Visual Impairment (VA <6/18)**						
50-59	43/379	117.9 (91.1-151.4)	76/542	136.8 (108.9-170.5)	119/921	128.9 (107.9-153.2)
60-69	77/301	272.0 (215.4-336.9)	85/286	309.5 (263.6-359.4)	162/587	290.5 (249.6-335.2)
70-79	74/127	584.3 (491.9-671.1)	55/101	542.0 (431.3-648.7)	129/228	565.3 (489.3-638.3)
≥80	19/31	622.1 (447.2-770.1)	20/32	627.0 (421.1-795.3)	39/63	624.5 (493.1-739.9)
All ages	213/838	258.5 (226.1-293.7)	236/961	246.2 (216.6-278.4)	449/1799	251.9 (228.5-276.8)
**Person Cataract Visual Impairment (VA <6/18)**						
50-59	22/399	53.5 (36.6-77.5)	29/552	51.5 (35.3-74.5)	51/951	52.4 (40.0-68.2)
60-69	42/325	131.6 (96.9-176.1)	50/310	160.1 (120.2-210.2)	92/635	145.7 (118.5-177.9)
70-79	42/155	276.9 (203.9-364.0)	43/135	319.1 (237.3-413.8)	85/290	296.8 (247.0-351.8)
≥80	19/43	432.3 (294.9-580.9)	20/45	457.4 (315.3-606.8)	39/88	445.2 (353.9-540.3)
All ages	125/922	134.2 (110.3-162.4)	142/1042	135.5 (112.1-162.9)	267/1964	134.9 (117.1-154.9)
**Either-Eye Severe Visual Impairment (VA <6/60)**						
50-59	24/391	63.8 (43.7-92.3)	26/555	46.4 (30.2-70.7)	50/946	53.8 (40.2-71.7)
60-69	46/324	142.4 (110.6-181.6)	45/315	149.6 (112.8-195.9)	91/639	146.0 (121.2-175.0)
70-79	40/157	260.3 (196.8-335.7)	39/134	297.2 (218.2-390.4)	79/291	277.5 (222.9-339.6)
≥80	25/55	461.0 (327.0-600.8)	19/55	362.0 (232.9-514.6)	44/110	410.7 (317.2-511.0)
All ages	135/927	146.4 (124.5-171.5)	129/1059	125.3 (104.5-149.5)	264/1986	135.1 (119.1-152.9)
**Person Severe Visual Impairment (VA <6/60)**						
50-59	5/404	13.3 (5.7-30.6)	7/563	13.0 (5.6-29.9)	12/967	13.1 (7.3-23.6)
60-69	21/348	57.6 (37.6-87.2)	26/332	82.4 (52.7-126.7)	47/680	69.9 (50.0-96.8)
70-79	26/181	154.6 (106.4-219.4)	27/155	169.0 (112.4-246.0)	53/336	161.3 (122.3-209.8)
≥80	17/63	260.8 (172.3-374.1)	11/64	171.4 (96.7-285.5)	28/127	215.2 (153.0-293.9)
All ages	69/996	69.2 (54.9-86.9	71/1114	64.3 (49.1-83.8)	140/2110	66.6 (54.9-80.6)
**Either-Eye Cataract Blindness (VA <3/60)**						
50-59	10/393	24.4 (11.6-50.6)	10/555	17.4 (9.3-32.3)	20/948	20.4 (12.8-32.4)
60-69	27/329	81.4 (56.1-116.6)	15/317	50.0 (30.0-82.0)	42/646	65.7 (49.4-86.9)
70-79	23/159	156.5 (97.0-242.7)	24/137	174.1 (119.8-246.2)	47/296	164.8 (122.6-217.8)
≥80	13/56	218.3 (127.6-347.7)	11/55	219.3 (116.2-375.1)	24/111	218.8 (141.4-322.6)
All ages	73/937	77.2 (58.4-101.4)	60/1064	58.1 (45.4-74.2)	133/2001	67.0 (55.6-80.6)
**Person Cataract Blindness (VA <3/60)**						
50-59	1/404	2.3 (0.3-16.8)	0/563		1/967	1.0 (0.1-7.2)
60-69	5/350	14.9 (6.1-35.7)	3/332	9.9 (2.9-32.9)	8/682	12.4 (6.0-25.4)
70-79	4/182	29.7 (10.7-79.6)	4/155	24.8 (9.8-61.4)	8/337	27.4 (13.7-54.1)
≥80	2/67	38.0 (8.4-155.7)	8/64	116.7 (58.4-219.7)	10/131	76.7 (40.7-140.1)
All ages	12/1003	13.8 (7.9-24.2)	15/1114	13.3 (8.1-21.9)	27/2117	13.6 (9.4-19.5)

Abbreviation: VA, visual acuity.

The types of lens opacities associated with the level of VI were examined (eTable 2 in the Supplement). The most common findings were mixed opacities followed by nuclear opacities only, cortical opacities only, and posterior subcapsular opacities only in all vision categories.

### Estimates of Incidence

A total of 2159 participants (49.5%; with a mean [SD] age of 62.5 [9.3] years and with 1140 men [52.8%]) were followed up in 2013 to 2014. Of these participants, 2129 (98.6%) had a complete examination, including lens status.

At baseline, 3591 participants were without visually significant cataract and 1821 (50.7%) were followed up, with 1799 (98.8%) receiving a complete lens examination and therefore at risk of developing incident visually impairing cataract. In the 6-year follow-up period, 449 (24.9%) of the 1799 participants who were at risk developed a visually significant cataract (VA <6/18 with the presence of a cataract), and 7 (1.5%) of these 449 had become cataract blind.

**Figure 1.**
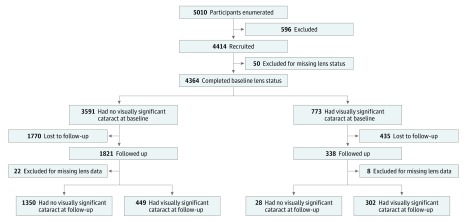
Study Participants Visually significant cataract indicates visual acuity less than 6/18 and proven cataract.

Overall, 1944 participants had a cataract on clinical examination at baseline, of whom 773 (39.8%) had a visually significant cataract at baseline, with proportionally fewer (330 [42.7%]) available for follow-up examination. Most of these individuals (302 [91.5%]) had a visually significant cataract at follow-up, whereas 28 (8.5%) no longer had a visually significant cataract at follow-up despite no report of an operation (Figure 1). Eighteen (6.3%) of 284 participants at baseline who were referred for cataract surgical procedure had undergone an operation at follow-up.

Because of the high percentage (50.5%) of people lost to follow-up, we compared baseline features between participants who were followed up and those who were not (eTable 3 in the Supplement). Notable differences were found between these 2 groups and those not known to be deceased (n = 1524 [42.4%]), including proportionally fewer Kikuyus and Kalenjins (the 2 major ethnic tribes in those not followed up) and proportionally more rural than urban dwellers among those who were followed up. Notable differences between those followed up and those known to be deceased included younger mean age (60.9 years vs 67.1 years), lower systolic blood pressure (139.1 mm Hg vs 145.1 mm Hg), lower random blood glucose (93.7 mg/dL vs 100.9 mg/Dl [to convert to millimoles per liter, multiply by 0.0555]), higher body mass index (10.4% vs 23.4% underweight at baseline), and lower alcohol consumption.

The 6-year cumulative incidence of visually significant cataract in either eye, after adjusting for those lost to follow-up using the inverse probability weighting model, was 251.9 (95% CI, 228.5-276.8) per 1000 for all ages, with an increase with age from 128.9 (95% CI, 107.9-153.2) per 1000 for the group aged 50 to 59 years, 290.5 (95% CI, 249.6-335.2) per 1000 for the group aged 60 to 69 years, 565.3 (95% CI, 489.3-638.3) per 1000 for the group aged 70 to 79 years, and 624.5 (95% CI, 493.1-739.9) per 1000 for the group aged 80 years or older (Table 1). This cumulative incidence equates to an annual incidence of visually significant cataract (<6/18 in either eye) of 45.0 per 1000 people aged 50 years or older, along with 2.5 per 1000 people per year in this age group developing cataract blindness (VA <3/60 in both eyes).

The 6-year incidence of persons (with better-seeing eye) becoming visually impaired was 134.9 (95% CI, 117.1-154.9) per 1000, severely visually impaired was 66.6 (95% CI, 54.9-80.6) per 1000, or blind was 13.6 (95% CI, 9.4-19.5) per 1000 from cataract (Table 1).

When the cumulative incidence was extrapolated to all people in Kenya aged 50 years or older, the estimated number of individuals per year who might develop visually impairing cataract in either eye was 148 280 (95% CI, 134 510-162 950), become visually impaired from cataract in the better-seeing eye was 86 690 (95% CI, 75 240-99 570), develop severely visually impairing cataract in either eye was 88 630 (95% CI, 78 140-100 280), become severely visually impaired from cataract in the better-seeing eye was 46 690 (95% CI, 38 500-56 480), develop cataract blindness in either eye was 44 260 (95% CI, 36 700-53 240), and develop cataract blindness in the better-seeing eye was 9540 (95% CI, 6610-13 750) (Table 2).

**Table 2.** Extrapolated Number of New Adults With Visually Significant or Blinding Cataract in 2015

Age Group, y	Extrapolated No. (95% CI)		
	Male	Female	Overall
**Either-Eye Cataract Visual Impairment (VA <6/18)**			
50-59	19 750 (15 250-25 350)	25 640 (20 410-31 950)	45 760 (38 330-54 390)
60-69	21 970 (17 400-27 210)	31 380 (26 730-36 450)	52 950 (45 490-61 080)
70-79	16 730 (14 080-19 210)	20 760 (16 520-24 840)	37 700 (32 630-42 570)
≥80	2910 (2090-3600)	4510 (3030-5720)	7360 (5810-8720)
All ages	68340 (59 780-77 650)	79 940 (70 320-90 390)	148 280 (134 510-162 950)
**Person Cataract Visual Impairment (VA <6/18)**			
50-59	9230 (6320-13 370)	9800 (6720-14 170)	18 990 (14 510-24 750)
60-69	11 570 (8520-15 490)	17 420 (13 070-22 860)	28 700 (23 340-35 030)
70-79	9760 (7190-12 830)	15 370 (11 430-19 930)	24 610 (20 480-29 180)
≥80	3080 (2100-4130	4970 (3430-6590)	7950 (6320-96,50)
All ages	39 110 (32130-47 320)	47 620 (39 410-57 240)	86 690 (75 240-99 570)
**Either-Eye Cataract and Severe Visual Impairment (VA <6/60)**			
50-59	10 950 (7500-15 830	8870 (5780-13 510)	19 500 (14 560-26 000)
60-69	12 380 (9610-15 780)	16 810 (12 670-22 010)	29 060 (24 120-34 820)
70-79	9560 (7230-12 330)	14 600 (10 720-19 190)	23 750 (19 080-29 060)
≥80	3840 (2720-5010)	4870 (3130-6920)	8860 (6840-11 020)
All ages	43 140 (36 670-50 520)	45 330 (37 800-54 110)	88 630 (78 140-100 280)
**Person Cataract Severe Visual Impairment (VA<6/60)**			
50-59	2330 (1000-5380)	2530 (1090-5790)	4850 (2690-8730)
60-69	5350 (3490-8100)	9670 (6180-14870)	14 690 (10 520-20 350)
70-79	6500 (4470-9220)	9420 (6270-13720)	15 730 (11 930-20 470)
≥80	2890 (1910-4150)	2860 (1610-4760)	5940 (4230-8120)
All ages	22 060 (17500-27700)	24 590 (18 780-32 030)	46 690 (38 500-56 480)
**Either-Eye Cataract Blindness (VA <3/60)**			
50-59	4190 (1990-8700)	3330 (1790-6170)	7400 (4640-11 750)
60-69	7150 (4930-10 250)	5650 (3400-9270)	13 190 (9920-17 450)
70-79	5800 (3590-8990)	8670 (5970-12 260)	14 260 (10 610-18 850)
≥80	1880 (1100-2990)	2990 (1580-5110)	4820 (3110-7100)
All ages	22 940 (17 350-30 140)	21 120 (16 490-26 960)	44 260 (36 700-53 240)
**Person Cataract Blindness (VA <3/60)**			
50-59	410 (60-2950)	0	370 (50-2670)
60-69	1390 (570-3330)	1170 (350-3880)	2620 (1280-5360)
70-79	1250 (450-3350)	1380 (540-3430)	2680 (1340-5290)
≥80	440 (100-1810)	1970 (980-3700)	2180 (1150-3970)
All ages	4430 (2520-7760)	5110 (3090-8390)	9540 (6610-13 750)

Abbreviation: VA, visual acuity.

**Figure 2.**
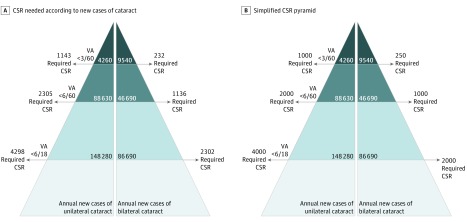
Estimated Minimal Cataract Surgical Rate (CSR) for Annual Incidence of Visually Impairing Cataract in Kenya The different surgical thresholds shown are based on presenting visual acuity (VA) in either the better or worse seeing eye.

These rates indicate that a CSR of 232 is required to match the annual new cases of persons who are cataract blind. This CSR goes up as the threshold for surgical procedures goes down (eTable 4 in the Supplement; Figure 2).

Multivariable analysis showed alcohol consumption, diabetes, educational level, and increasing age to be associated with incident visually impairing cataract. With an RR of 1.4 (95% CI, 1.1-1.8) in current alcohol drinkers, compared with never drinkers, former drinkers were not at an increased risk (RR, 1.1; 95% CI, 0.9-1.3). Those with diabetes had an RR of 1.7 (95% CI, 1.3-2.3) compared with those without diabetes, and those with higher educational level tended to have less incident cataract (primary education only, 341.2 [95% CI, 299.8-385.3] vs more than secondary education, 91.2 [95% CI, 55.7-145.7]). Compared with those aged 50 to 59 years, the RR was 2.0 (95% CI, 1.6-2.6) in those aged 60 to 69 years, 3.7 (95% CI, 2.9-4.7) in those aged 70 to 79 years, and 3.8 (95% CI, 2.6-5.5) in those aged 80 years or older (Table 3).

**Table 3.** Age-Adjusted and Multivariable Analysis in the Nakuru Eye Disease Cohort Study

Variable	Study Sample (n = 1799)				
	At Risk of Cataract, No. (%)	Incident Cataract, No. (%)	Risk per 1000/6 y (95% CI)	Risk Ratio (95% CI)	
				Age Adjusted	Multivariable Adjustment
Age, y					
50-59	921 (51.2)	110 (6.1)	119.0 (98.0-143.8)	1 [Reference]	1 [Reference]
60-69	587 (32.6)	152 (8.4)	270.3 (231.6-312.8)	2.3 (1.8-2.9)	2.0 (1.6-2.6)
70-79	228 (12.7)	122 (6.8)	534.1 (458.5-608.2)	4.5 (3.5-5.7)	3.7 (2.9-4.7)
≥80	63 (3.5)	38 (2.1)	601.3 (459.2-728.2)	5.1 (3.6-7.1)	3.8 (2.6-5.5)
Sex					
Male	838 (46.6.)	204 (11.3)	246.7 (215.7-280.6)	1 [Reference]	NA
Female	961 (53.4)	218 (12.1)	226.0 (198.1-256.6)	1.0 (0.9-1.2)	NA
BMI classification^a^					
Underweight	187 (10.4)	61 (3.4)	329.6 (262.3-404.6)	1 [Reference]	NA
Normal	890 (49.5)	231 (12.8)	260.7 (228.6-295.7)	0.9 (0.7-1.1)	NA
Overweight	444 (24.7)	86 (4.8)	193.7 (157.5-235.9)	0.8 (0.6-1.0)	NA
Obese	272 (15.1)	42 (2.3)	155.0 (111.1-212.0)	0.6 (0.4-0.9)	NA
Location					
Rural	1332 (74.0)	333 (18.5)	259.0 (232.0-288.0)	1 [Reference]	NA
Urban	467 (26.0)	89 (4.9)	192.3 (158.6-231.3)	0.9 (0.8-1.2)	NA
SES quartile^a^					
Lower	378 (21.0)	123 (6.8)	329.7 (282.0-381.2)	1 [Reference]	NA
Lower middle	491 (27.6)	124 (6.9)	253.8 (215.5-296.2)	0.8 (0.7-1.0)	NA
Upper middle	476 (26.5)	107 (5.9)	224.8 (187.1-267.7)	0.8 (0.7-1.0)	NA
Upper	446 (24.8)	68 (3.8)	155.6 (129.2-186.3)	0.6 (0.5-0.8)	NA
Smoking status					
Never smoked	1255 (69.8)	284 (15.8)	228.6 (203.1-256.2)	1 [Reference]	NA
Former smoker	138 (7.7)	37 (2.1)	259.3 (190.2-342.8)	1.0 (0.8-1.4)	NA
Current smoker	406 (22.6)	101 (5.6)	250.6 (209.9-296.2)	1.0 (0.8-1.1)	NA
Hypertensive^a^					
No	917 (51.0)	204 (11.3)	226.3 (199.9-255.1)	1 [Reference]	NA
Yes	875 (48.6)	216 (12.0)	244.2 (214.0-277.2)	0.9 (0.8-1.1)	NA
Diabetes^a^					
No	1710 (95.0)	388 (21.6)	227.3 (204.8-251.6)	1 [Reference]	1 [Reference]
Yes	88 (4.9)	34 (1.9)	388.8 (302.2-483.1)	1.6 (1.2-2.0)	1.7 (1.3-2.3)
Alcohol use status^a^					
Never drank	774 (43.0)	155 (8.6)	197.6 (168.9-229.8)	1 [Reference]	1 [Reference]
Former drinker	753 (41.9)	190 (10.6)	256.1 (224.8-290.1)	1.1 (0.9-1.3)	1.1 (0.9-1.3)
Current drinker	269 (15.0)	77 (4.3)	279.9 (220.0-348.8)	1.3 (1.0-1.7)	1.4 (1.1-1.8)
Ethnic group					
Kikuyu	1172 (65.1)	278 (15.5)	238.9 (212.0-268.0)	1 [Reference]	NA
Kalenjin	419 (23.3)	108 (6.0)	266.1 (226.1-310.3)	1.2 (1.0-1.5)	NA
Other	208 (11.6)	36 (2.0)	182.8 (137.8-238.4)	1.1 (0.8-1.4)	NA
Educational level^a^					
No education	180 (10.0)	19 (1.1)	105.3 (68.5-158.3)	1 [Reference]	1 [Reference]
Primary	494 (27.5)	167 (9.3)	341.2 (299.8-385.3)	2.0 (1.3-3.1)	2.2 (1.4-3.4)
Secondary	922 (51.3)	219 (12.2)	241.2 (211.7-273.4)	1.7 (1.1-2.7)	1.9 (1.2-2.9)
College/university	202 (11.2)	17 (0.9)	91.2 (55.7-145.7)	0.9 (0.5-1.7)	0.9 (0.5-1.8)

Abbreviations: BMI, body mass index; NA, not applicable; SES, socioeconomic status.

^a^ These variables were missing data: BMI had 6 missing values; SES, 8; hypertensive (yes or no), 7; diabetes (yes or no), 1; alcohol status, 3; and educational level, 1.

## Discussion

To our knowledge, this study is the first long-term population-based survey on eye disease in Africa. The annual incidence of visually impairing cataract (VA <6/18 in either eye) in those aged 50 years or older was 45.0 per 1000 people per year and 2.5 per 1000 per year were cataract blind (VA <3/60 in both eyes).

Increasing age, diabetes, alcohol consumption, and low educational level were associated with incident visually impairing cataract. Aging has been a well-described risk factor for incident cataract throughout the world.^12,13,24,25^ Diabetes has also been associated with incident cataract,^26,27^ although most cohort studies have not found an association with alcohol consumption; however, a U-shaped association was found in an Australian cohort, with moderate consumption being seemingly protective compared with abstinence or heavy consumption.^28^ Some evidence of an inverse association exists between educational level and incident cataract,^29,30^ as demonstrated in this population: notably, educational level affects incidence of cataract operation more commonly than do cataract formation.^31^

Previous studies have reported that, at baseline, 63 of the 71 who were blind were known to have cataract.^5^ At the 6-year follow-up, 2164 participants were seen with complete follow-up data, of whom 24 were blind at baseline and therefore were excluded from the analysis because they were not considered at risk of becoming blind; in total, 29 new cases of bilateral blindness were confirmed, which equated to a 6-year cumulative incidence of VI of 11.9% (95% CI, 10.3%-13.8%) and blindness of 1.51% (95% CI, 1.0%-2.2%).^5^ In this analysis, we found that most incident VI and blindness cases were associated with cataract.

Of the 29 blind persons at 6-year follow-up, 27 had bilateral cataracts. However, blindness could not be associated with a single condition when a participant had comorbidity, and this finding should be kept in mind when interpreting the data. Six-year cumulative incidence of cataract-associated blindness was 13.6% (95% CI, 9.4%-19.5%), which took into account the definition used in this cohort that a person was deemed an incident case if found to be pseudophakic at the 6-year follow-up, assuming the person had visually impairing cataract between assessments that had warranted surgical intervention. The exact proportion of overall incident blindness that can be associated with cataract was not possible, but cataract was the primary risk factor of incident blindness.

Past analysis of this cohort with regard to the incidence of diabetes and diabetic retinopathy^7^ showed cataract as a growing public health concern and diabetes as a risk factor for cataract. In this cohort, given the high prevalence and incidence of cataract, cataract, not diabetic retinopathy, was the leading risk factor in VI in diabetes.

Blindness and VI from cataract are associated with reduced quality of life^32^ and visual function, which can be reversed by low-cost surgical management.^33^ Considerable social and economic disadvantages are associated with cataract, especially in low-income communities, which may perpetuate the cycle of poverty.^34^ Conversely, poverty can be alleviated with the provision of cataract surgery.^35^ Management of cataract is recognized as a priority for the VISION 2020: The Right to Sight global initiative,^36^ which targets avoidable blindness. However, to our knowledge, incidence data including risk factors for visually impairing cataract were not previously available from the African continent, limiting the ability to effectively plan and to resource services for the continent.

On the basis of presenting VA less than 6/18 in either eye with a cataract verified by dilated slitlamp examination or the participant being newly pseudophakic, we found the incidence of cataract in Kenya to be high. As expected, the incidence of visually impairing cataract increased substantially with age. Comparison with other cohorts is limited, in part because of the lack of other data from the region and variations on the definition of visually impairing cataract; however, the estimates from the Nakuru Eye Disease Cohort Study show a higher incidence than in most other cohort studies outside of Kenya.^30,31,37,38,39^ This higher incidence may be associated with the study population’s high exposure to UV light as well as genetic and nutrition factors. The high prevalence of untreated cataract in the Nakuru Eye Disease Cohort Study may reflect a combination of limited access to ophthalmic services and high incidence of new cataract.^40^

This study also highlighted the low uptake of services by those needing cataract operation. At baseline, all participants identified by the lead ophthalmologist as having an operable cataract were offered a referral to the regional eye unit. However, few participants accessed the service, with only 18 (6.3%) of 284 individuals at follow-up reporting to have had surgical intervention. Barriers to cataract surgical procedures have been previously described in this population and included lack of awareness, high cost, distance from services, fear, and feeling that treatment was unnecessary.^41,42^ Ultimately, these barriers meant that visually significant cataract remained untreated.^43^

The results suggested that 148 280 new cases of eyes with VI (VA <6/18) per year existed, owing to cataract in people aged 50 years or older, of whom 9540 were blind. Extrapolating these estimates suggested that either 232 (only 1 eye of people who had VA <3/60 in both eyes), 2305 (all eyes with VA <6/60 with cataract), or 4298 (all eyes with VA <6/18 with cataract) cataract operations needed to be conducted per million population per year (CSR) to manage the new cataract cases, depending on which vision threshold for surgical intervention was used.

### Strengths and Limitations

Strengths of the Nakuru Eye Disease Cohort Study included being a representative population-based sample in an area of ethnic, socioeconomic, and educational diversity; having a large sample size; undertaking a comprehensive assessment of risk factors; providing high-quality assessment of vision, and using the same tools at baseline and follow-up. The methods it used to assess ophthalmic disease were consistent with those in studies of well-developed health systems in high-income countries, such as the United States^44^ and Australia,^45^ that used the latest available equipment.^14^

A limitation of this study was the low-participation rate at follow-up (50%); however, having the baseline characteristics of nonparticipants was a strength that enabled weighting, which ensured better estimates of cumulative incidence. This information on those lost to follow-up may have led to an underestimation or overestimation of incident cataract VI and blindness, depending on the general characteristics of the nonrespondents. The predominant risk factor for incident VI or blindness was age, and given that age was closely matched between participants and nonparticipants (mean [SD] age, 62.7 [9.4] years vs 62.5 [10.4] years), the estimates were likely to be an acceptable reflection. This is further supported by minimal changes being apparent after adjusting estimates for missing data.

Reasons for the low participation included ethnic violence, which displaced large numbers of people in the study sample area, and postelection violence in 2007 and 2008, which led to the internal displacement of up to 600 000 people and to 1300 fatalities.^46^ In numerous study clusters, entire ethnic groups present at baseline were no longer available or traceable at follow-up. Great efforts were made to locate individuals on 2 or 3 preexamination visits. We promoted attendance by providing transportation support and notification of alternative dates to attend clinics in the same location.

Another study limitation included the restriction of the inclusion criteria at baseline to those 50 years or older, which reduced the generalizability of the results to the entire population. This restriction is, however, comparable with most population-based studies of eye disease, which limit inclusion to 40- or 50-year-old participants. Sampling people aged 50 years or older was appropriate for the outcomes of interest in this study, given that the highest prevalence and incidence of cataract were in this age group, making this sample appropriate both for epidemiologic (sample size considerations) as well as for public health and policy planning purposes.^47^ The definitions of blindness and VI in this study were based solely on presenting central logMAR VA and did not include peripheral vision loss. The definitions potentially underestimated incident VI and blindness when compared with studies that included these criteria, although this was of less concern given that the focus of the study was cataract.

The current estimate of CSR in Kenya is 550 per 1 million population. Recent estimates indicate that 100 ophthalmologists work in a country of approximately 45 million, and 50% of these ophthalmologists are based in the capital city of Nairobi. This lack of eye care practitioners leaves 92% of the population (approximately 40 million people) under the care of 50 ophthalmologists. Overall, Kenya is better than many other African countries in terms of human and other resources, despite still being well below recommended targets.^48^ Continued efforts to strengthen the eye health system in Kenya are necessary to support the growing unmet need of this aging and growing population. High-quality, high-volume surgical treatment for cataract and greater awareness of and demand for eye health services at the community level are also required.

## Conclusions

The incidence of visually impairing cataract in this population of Kenyan adults was considerably higher than in comparable studies worldwide. Cataract remains the priority condition for the prevention of avoidable blindness and VI. High-quality, high-volume cataract operations and an increased awareness and demand for services at the community level are required to lower the burden of VI and blindness.

## Notes

Supplement. **eMethods.** Method Details **eTable 1.** Definitions of Incidence **eTable 2.** Types of Lens Opacity at Baseline Among Those Where Main Cause of VI Is Cataract **eTable 3.** Baseline Characteristics of All Individuals With Non-visually Significant Cataract at Baseline According to Availability of Visually Significant Cataract Status at Six-year Follow Up **eTable 4.** Cataract Surgical Rates (CSR) Required to Match Incident Cataract Vision Loss at Different Thresholds
