# Supplementary material for: Incidence of Visually Impairing Cataracts Among Older Adults in Kenya
Source: JAMA Netw Open. 2019 Jun 28;2(6):e196354. doi: 10.1001/jamanetworkopen.2019.6354 (PMC6604086; doi:10.1001/jamanetworkopen.2019.6354)
Supplement: Supplement. — eMethods. Method Details eTable 1. Definitions of Incidence eTable 2. Types of Lens Opacity at Baseline Among Those Where Main Cause of VI Is Cataract eTable 3. Baseline Characteristics of All Individuals With Non-visually Significant Cataract at Baseline According to Availability of Visually Significant Cataract Status at Six-year Follow Up eTable 4. Cataract Surgical Rates (CSR) Required to Match Incident Cataract Vision Loss at Different Thresholds [file jamanetwopen-2-e196354-s001.pdf]

Supplementary Online Content

Bastawrous A, Mathenge W, Nkurikiye J, et al. Incidence of visually impairing cataracts among older adults in Kenya. *JAMA Netw Open.* 2019;2(6):e196354. doi:10.1001/jamanetworkopen.2019.6354

eMethods. Method Details

eTable 1. Definitions of Incidence

eTable 2. Types of Lens Opacity at Baseline Among Those Where Main Cause of VI Is Cataract

eTable 3. Baseline Characteristics of All Individuals With Non-visually Significant Cataract at Baseline According to Availability of Visually Significant Cataract Status at Six-year Follow Up

eTable 4. Cataract Surgical Rates (CSR) Required to Match Incident Cataract Vision Loss at Different Thresholds

This supplementary material has been provided by the authors to give readers additional information about their work.

©2019 Bastawrous A et al. *JAMA Network Open*

eMethods

Additional ethical considerations

Baseline approval was provided by the Kenya Medical Research Institute Ethics Committee and by the African Medical and Research Foundation (AMREF) Ethics Committee, Kenya for the follow-up (AMREF-ESRC P44/12). For both phases approval was granted by the Rift Valley Provincial Medical Officer and the Nakuru District Medical Officer of Health. Approval was sought from the administrative heads in each cluster, usually the village chief. They were also given a copy of the consent form to read and pass on to those in the village.

Retracing at Follow-up - Advance Team

Approximately one week before the follow-up examination clinic was planned for a given cluster, a field officer studied the maps of the village including GPS coordinates recorded at baseline and made phone contact with the village chief or guide to arrange the visit. At the planning visit, a list of study participants were given to the chief and a local village guide was recruited to assist location of the study participants. At this visit the examination site was established and identification of amenities such as electricity, water and road access were made. Two days prior to the clinic, the field officer reminded chiefs of the visit by phone and notified them and the guide of the advance team's arrival.

On the day prior to the examination clinic, the Advance Team visited homes of baseline participants and confirmed their identity using National Identity cards and invited them to attend the examination clinic the following day. All identified participants were also asked to help locate baseline participants that had not been found.

Anthropometry

A nurse performed and recorded measures of participants: height (Leicester Height Measure, Chasmors Ltd, London); weight (Seca 761 Medical Class 4 Scales mechanical ground scale, Williams Medical Supplies, London); waist and hip circumference (Chasmors WM02 Body Tape measure), and three measures of blood pressure (Omron® Digital Automatic Blood Pressure Monitor Model HEM907), each ten minutes apart. In addition, at follow-up, bioimpedence (Tanita Segmental Body Composition Monitor) was performed.

At baseline, capillary blood was taken from all participants for random blood glucose, in addition at follow-up glycosylated haemoglobin was taken in all with a self-reported history of diabetes, or random blood glucose of ≥7.0mmol/L and a further 10% of non-diabetics (based on history and random blood glucose).

Interview

An interviewer performed a structured interview in the participant's preferred language covering i) demographic details including; name, year of birth, ethnicity and education level; ii) past medical and ocular history including medical or ophthalmic medication or surgery and relevant family history; iii) known risk factors, including smoking and tobacco consumption and alcohol intake; iv) socioeconomic status based on job, housing conditions, ownership of material goods and livestock which is translated in to a score based on previous work in the same population.[17]

Visual Acuity Definitions

WHO definitions of visual impairment and blindness were used throughout [19]. Monocular visual impairment is defined as visual acuity <6/18 (20/60) in either eye. Visual impairment is

defined as a visual acuity of <6/18 in the better eye. Monocular blindness was defined as a visual acuity of <3/60 (20/400) in either eye. A person was considered to be blind if the visual acuity in the better eye was <3/60. The definition of visual impairment also includes those who were blind.

Diabetes Definition

Diabetes was defined as (1) Self-reported in the history, or (2) random glucose of ≥11.0mmol/L, or (3) HbA1c ≥7.0.

Extrapolation of data

Estimates of cumulative incidence were extrapolated to estimate the number of adults over the age of 50 with incident visual impairment or blindness in Kenya. This was calculated by taking 2015 population estimate from Kenya (Census Bureau of Kenya) by age category and gender and multiplying this by the age and gender specific estimates of annual cumulative incidence.

©2019 Bastawrous A et al. *JAMA Network Open*

eTable 1. Definitions of Incidence

Category	At risk	Incident case
Eye with incident visually impairing cataract	Neither eye had both a cataract and VA ≥6/18	Either eye became <6/18 and had confirmed cataract or was incident pseudophakic
Person with incident visually impairing cataract	Person was ≥6/18 in the better eye	Person became <6/18 and had confirmed cataract or was incident pseudophakic
Eye with incident blinding cataract	Neither eye had both a cataract and VA was ≥3/60	Either eye became <3/60 and had confirmed cataract
Person with incident blinding cataract	Person was ≥3/60 in the better eye	Person became <3/60 and had confirmed cataract

©2019 Bastawrous A et al. *JAMA Network Open*

eTable 2. Types of Lens Opacity at Baseline Among Those Where Main Cause of VI Is Cataract

Visual Acuity	Nuclear Only	Cortical Only	PSC Only	Mixed	Total Numbers
Moderate VI (<6/18-6/60)	21(15.3%)	18(13.1%)	2(1.5%)	96(70.1%)	137(100%)
Severe VI (<6/60-3/60)	3(27.3%)	0	0	8(72.7%)	11(100%)
Blind (<3/60)	5(15.6%)	2(6.3%)	0	25(78.1%)	32(100%)
Total prevalence	29(17.6%)	20(11.7%)	2(1.0%)	129(69.8%)	180(100%)

PSC= Posterior subcapsular cataract

eTable 3. Baseline Characteristics of All Individuals With Non-visually Significant Cataract at Baseline According to Availability of Visually Significant Cataract Status at Six-year Follow Up (N=3,591)

			Participants	Non-participants or not included in analysis			
**Baseline Characteristics**		*Missing Values*	*Followed-up n=1,799 (50.1%)*	*Not followed-up Alive/Unknown/ cataract status missing n=1,524 (42.4%)*	*p-value**	*Deceased n=268 (7.5%)*	*p-value***
Age in years, mean (SD)		0	60.9 (8.2)	60.4 (8.7)	0.259	67.1 (11.5)	<0.001
Systolic BP in mmHg, mean (SD)		17	139.1 (23.3)	139.5 (24.0)	0.628	145.1 (29.3)	0.001
Diastolic BP in mmHg , mean (SD)		17	83.2 (13.1)	83.5 (13.2)	0.551	83.3 (16.2)	0.884
Random Blood Glucose, mean (SD)		76	5.2 (2.3)	5.3 (2.1)	0.198	5.6 (3.4)	0.013
Sex, % (n)	Female	0	838 (46.6%)	699 (45.9%)	0.720	153 (57.1%)	0.001
	Male		961 (53.4%)	825 (54.1%)		115 (42.9%)	
BMI, % (n)	Underweight (<18.5kg/m2)	24	187 (10.4%)	185 (12.3%)	0.269	62 (23.4%)	<0.001
	Normal (18.5-24.99kg/m2)		890 (49.6%)	717 (47.5%)		127 (47.9%)	
	Overweight (25-29.99kg/m2)		444 (24.8%)	377 (25.0%)		51 (19.2%)	
	Obese (30+kg/m2)		272 (15.2%)	230 (15.2%)		25 (9.4%)	
Vision status impaired (<6/12 better eye), % (n)	Normal	0	1774 (98.6%)	1505 (98.8%)	0.749	262 (97.8%)	0.265
	Impaired		25 (1.4%)	19 (1.2%)		6 (2.2%)	
Tribe, % (n)	Kikuyu	0	1172 (65.1%)	918 (60.2%)	<0.001	190 (70.9%)	0.105
	Kalenjin		419 (23.3%)	281 (18.4%)		54 (20.1%)	
	Other		208 (11.6%)	325 (21.3%)		24 (9.0%)	
Education, % (n)	None	8	180 (10.0%)	193 (12.7%)	0.049	24 (9.0%)	0.010
	Primary		494 (27.5%)	428 (28.2%)		98 (36.6%)	
	Secondary		922 (51.3%)	702 (46.3%)		120 (44.8%)	
	Higher		202 (11.2%)	194 (12.8%)		26 (9.7%)	
Residence, % (n)	Rural	0	1332 (74.0%)	824 (54.1%)	<0.001	198 (73.9%)	0.961
	Urban		467 (26.0%)	700 (45.9%)		70 (26.1%)	
SES Quartile, % (n)	Lower	22	378 (21.1%)	332 (22.0%)	0.010	74 (27.6%)	0.088
	Middle lower		491 (27.4%)	327 (21.7%)		69 (25.7%)	
	Middle upper		476 (26.6%)	390 (25.8%)		72 (26.9%)	
	Upper		446 (24.9%)	461 (30.5%)		53 (19.8%)	
Smokers, % (n)	Never	0	1255 (69.8%)	1112 (73.0%)	0.007	166 (61.9%)	0.036
	Former		138 (7.7%)	131 (8.6%)		26 (9.7%)	
	Current		406 (22.6%)	281 (18.4%)		76 (28.4%)	
Alcohol, % (n)	Never	11	774 (43.1%)	620 (40.9%)	0.031	83 (31.0%)	<0.001
	Former		753 (41.9%)	614 (40.5%)		130 (48.5%)	
	Current		269 (15.0%)	282 (18.6%)		55 (20.5%)	

*P-value for association between the baseline characteristic and the odds of having a valid cataract observation at follow up, amongst all participants identified as not having a visually significant cataract at baseline and not known to be deceased at follow up.

** P-value for association between the baseline characteristic and the odds of dying during the follow up period, amongst all participants identified as not having a visually significant cataract at baseline and either followed up or known to be

©2019 Bastawrous A et al. *JAMA Network Open*

eTable 4. Cataract Surgical Rates (CSR) Required to Match Incident Cataract Vision Loss at Different Thresholds

Category	Threshold of cataract	Cumulative six-year incidence / per 1,000 people (95%CI)	Extrapolated number of adults over 50 in Kenya per year	CSR (cataract operations per million per year)
Person blind	best eye sees <3/60	13.6 (9.4-19.5)	9,540	232
Eye blind	worst eyes sees <3/60	67.0 (55.6-80.6)	44,260	1,143
Person Severely Visually Impaired ≤6/60	best eye sees ≤6/60	66.6 (54.9-80.6)	46,690	1,136
Either eye Severely Visually Impaired ≤6/60	worst eye sees ≤6/60	135.1 (119.1-152.9)	88,630	2,305
Person visually impaired	best eye sees ≤6/18	134.9 (117.1-154.9)	86,690	2,302
Eye visually impaired	worst eyes sees ≤6/18	251.9 (228.5-276.8)	148,280	4,298

©2019 Bastawrous A et al. *JAMA Network Open*
